# Location-independent leadership: managers’ experiences leading prehospital emergency care in Sweden – a qualitative study

**DOI:** 10.1186/s12913-025-12433-1

**Published:** 2025-02-17

**Authors:** Henrik Lindlöf, Carl Savage, Karin Pukk Härenstam, Veronica Vicente

**Affiliations:** 1 Department of Clinical Science and Education, Södersjukhuset, Karolinska Institute, Stockholm, Sweden; 2 Department of Learning, Informatics, Management and Ethics, Medical Management Centre, Karolinska Institute, Stockholm, Sweden; 3 Academic EMS in Stockholm, Stockholm, Sweden; 4 Ambulance Medical Service in Greater Stockholm, Stockholm, Sweden; 5 Ambulance Medical Service in Västmanland, Västerås, Sweden; 6 Pediatric Emergency Department, Astrid Lindgren’S Children’S Hospital, Karolinska University Hospital, Stockholm, Sweden

**Keywords:** Prehospital emergency care, Management, Leadership, Context, Distance, Trust, Experience

## Abstract

**Introduction:**

Research into management and leadership in healthcare has revealed that the organizational context influences quality improvement, which is why research is needed to better understand the particulars of leadership in the relatively unexplored field of prehospital emergency care. This includes aspects of managerial work related to managers' experiences and their understanding of their roles and existing operational routines. Therefore, this study aims to explore managers' experiences with management and leadership in the context of prehospital emergency care in Sweden.

**Methods:**

A qualitative interview study design was conducted with 15 unit managers in prehospital emergency care from four of Sweden’s 21 regions. The transcripts from the in-depth interviews were subjected to inductive content analysis and reported according to the Consolidated Criteria for Reporting Qualitative Research.

**Results:**

Three generic categories related to the managerial role were identified: challenges, openness and trust, and experience-based leadership. The challenges managers faced were a. lack of physical proximity; b. staff needs for knowledge and competency development; c. staff level of responsibility related to operating procedures and guidelines; and d. work culture. Trust was developed through open and personalized communication, trusting relationships, authenticity, and empathic ability. Experience-based leadership was cultivated over time through practice, reflection, guidance from peers, theoretical leadership training, and the adaptation of organizational structures.

**Conclusions:**

We found the managerial role to be location independent, characterized by openness and trust, and cultivated through experience. In an environment characterized by academic training and work at distances, leaders manage individuals and remote teams while respecting individuals’ independence. They supported staff competence development and their desire to take responsibility through open and trusting relationships established through creating opportunities for competency development and a “learning-by-doing” epistemology built upon reflective practice.

**Supplementary Information:**

The online version contains supplementary material available at 10.1186/s12913-025-12433-1.

## Introduction

A growing body of research suggests that quality improvement in healthcare depends not only on the knowledge and skills of the individual healthcare provider but also on the organizational context in which the healthcare provider operates [1, 2]. In this context, management and leadership have been identified as key factors [1, 3, 4]. They could also be important for quality improvement in the area of prehospital emergency care (PHEC), especially as knowledge of and a culture of innovation and quality improvement are limited in this setting [5]. Management and leadership are shaped by contextual factors such as organizational culture, goals, structure and staff composition [6], task characteristics, physical distance, and time pressures [7]. For optimal effectiveness, organizations need both, as they tend to complement each other. We have chosen to see “management” as related to organizational objectives and processes, planning and structuring; “leadership” as related to social influence, establishing motivation and a long-tern vision for change [8, 9].

Research suggests that different leadership practices can impact health system performance, but they need to be adapted for use in healthcare contexts [10]. A better understanding of the situations that managers in PHEC face may help managers select or tailor leadership models and practices commonly linked to health care settings to achieve an optimum effect within PHEC. A commonly described model, transformational leadership, focuses on achievement, self-actualization, and the well-being of the organization through the leader’s charisma, inspiration, and intellectual stimulation [11]. Among nurses, this model has been positively linked to job satisfaction, unit performance, organizational climate and commitment, and employee turnover [10]. Adapting transformational practice to healthcare seems to require an understanding of follower expectations and context-specific norms, as well as an understanding of the degree of impact of staff education levels, where this type of leadership appears to matter more for nonprofessional than professional staff [10]. Authentic leadership, i.e., being true to oneself, acting on values and beliefs, and openly interacting with others has been shown to have a positive effect on management trust, engagement in the workplace, and patient outcomes [12]. Adaptive leadership, with a focus on learning, seems well suited to address the variation and complexity of certain health care contexts [13]. Collaborative and distributed leadership, in which healthcare professionals cooperate and share ownership and responsibility for tasks, can have a positive influence on organizational performance [14]. Servant leadership seems particularly suited to the healthcare context [15], as it can subscribe to the ethos of developing a shared culture of serving others [16]. Servant leadership has an indirect effect on organizational performance by allowing individuals to reach their full potential through being empowered to manage tasks. Like servant leadership is supportive leadership, where the leader communicates, interacts, and provides social and emotional assistance to care for followers [17]. Supportive leadership is the most preferred and used leadership style of nurse leaders and is positively correlated with staff job satisfaction [18].

PHEC addresses “immediate medical interventions taken by out-of-hospital health professionals”, in which ambulance care [19] is central [20]. In Sweden, each of the twenty-one regions is responsible for providing a plan for ambulance care in its geographical area, showing how it is to be organized [19]. Operational responsibilities are held by operation managers and are regulated by the Swedish Health and Medical Services Act (2027:30) [21]. The work of ambulance care is complex, involving difficult, often insecure environments that require specific types of knowledge to care for patients in unique, urgent, and nonurgent situations [22]. To achieve organisational and operational excellence, there is a need to know what kind of knowledge is desired in the field. Previous research has suggested that this includes medical care, contextual aspects, and nursing [22]. The care moment in the field includes advance information and staff expectations about what to care for, prior knowledge of the event, maintaining openmindedness, being prepared for the unprepared, and adapting to changing circumstances [23]. Standard operating procedures and guidelines to support clinical work and medical decisions exist, but they are not always sufficient or suitable for all missions [24]. There is a need to explore how management and leadership strategies can support staff’s work with patients and what contextual factors influence decision-making in the field [24].

Research on the influence of organisational contextual factors, management, leadership practices, and PHEC suggests that there is more to learn about effective leadership and management specific to PHEC. With a better understanding of the aspects and details of managerial experiences, work, and roles in this specific context with its operational routines, potential efforts towards improvement can be better tailored for effect. Therefore, this study aims to explore managers' experiences with management and leadership within PHEC in Sweden.

## Methods

### Design

We used a phenomenological approach and a qualitative interview study design [25]. Data were collected through in-depth interviews [26] and subjected to inductive content analysis [27]. We reported the results according to the Consolidated Criteria for Reporting Qualitative Research (COREQ) checklist (Appendix 1) [28].

### Setting

The study was conducted in four publicly funded PHEC units in four of Sweden's 21 regions, located in central Sweden, with different geographical sizes but similar mixes of urban and rural environments, population sizes, and organisational sizes and structures. In 2022, PHEC in these regions served a population of 1.3 million [29] and conducted 168,000 missions. Together, the regions had twenty-four unit managers, 750 employees (52% men, 48% women), and seventy-three ambulances (personal communication, operations managers in participating regions, 10 September 2023).

### Participants 

Through purposive sampling [30, 31], we sought to include unit managers with at least one year of PHEC management experience. Via the operating managers of the participating regions, we contacted seventeen unit managers via email. Two declined due to time pressures, resulting in fifteen study participants.

### Data collection 

The data were collected through in-depth one-to-one interviews with open-ended questions. The main interview question was “*What is your experience of management and leadership in prehospital emergency care?”* Follow-up questions such as *"Can you tell me more?"* were posed (Appendix 2). The interviews were developed for this particular study. They were conducted online via Microsoft Teams between April and October 2022 and were digitally recorded. The interviews lasted for an average of 53 min. The study sought to achieve pragmatic saturation, “a definition of saturation that stays true to foundational assumptions of grounded theory”, which can, however, never be absolute [32, 33].

### Data analysis

**Fig. 1:**
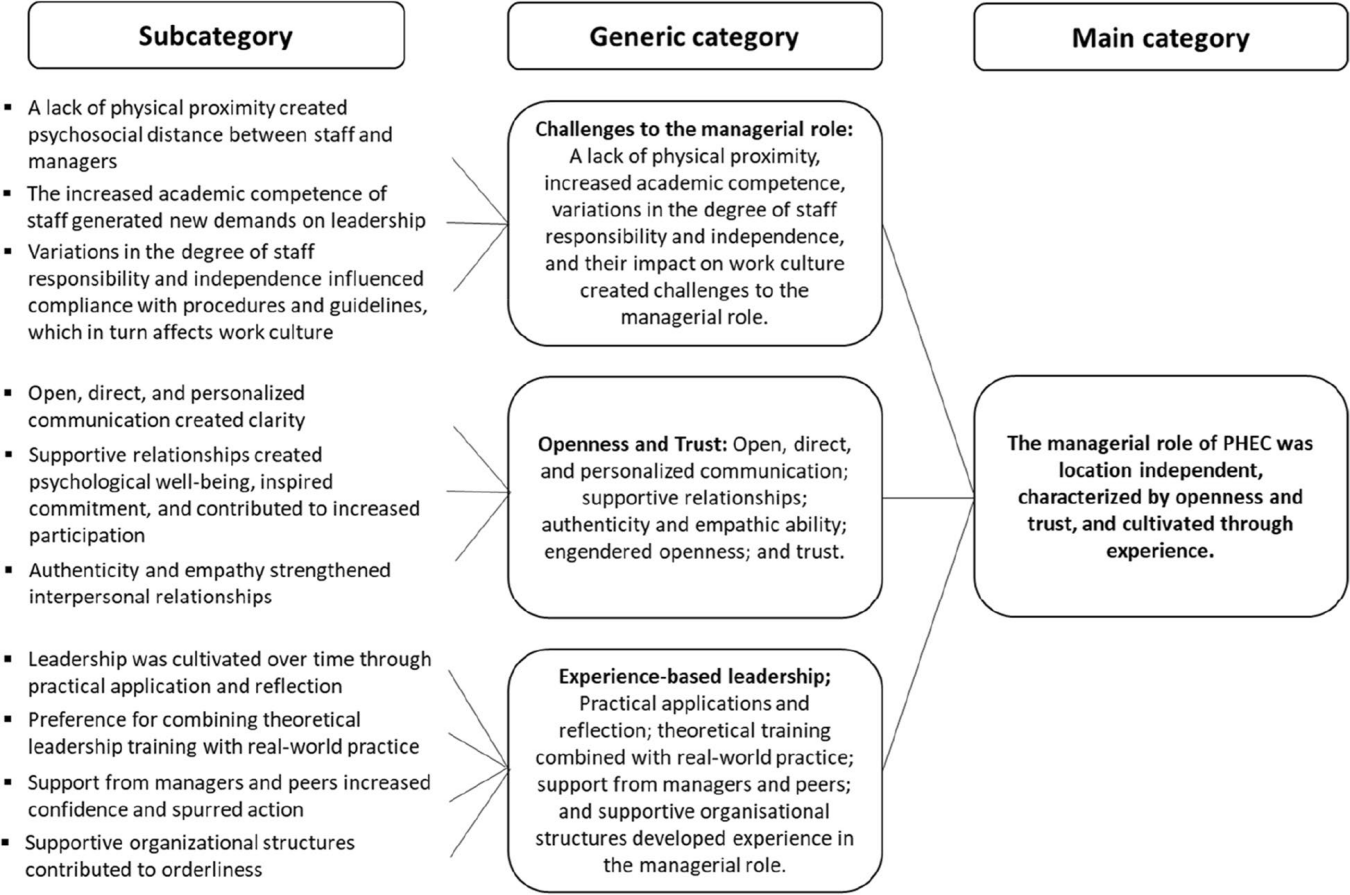
Experiences of leading prehospital emergency care

The interview data were subjected to inductive qualitative content analysis in accordance with the three-phase process (preparation, organizing, reporting) described by Elo and Kyngäs [27]. The data were managed via Microsoft Word software. In the preparation phase, interviews were listened to, transcribed verbatim, and read several times to develop a deep understanding of the content. In the organizing phase, a manifest focus was placed on the innate characteristics of the data [34], including textual processing of meaning-bearing units and codes, closely related to the aim. Gradually, the processing moved to a more latent level, with summaries of text and interpretations, to assign a meaning to the identified information [34]. In the reporting phase, the results were systematically described through subcategories and generic categories and summarized in one main category (Fig. 1). In this phase, quotations from different participants were added to illustrate important categories and to increase transparency, which contributes to study trustworthiness [35, 36]. A researcher whose native language was English reviewed the quotation translations.

## Results

### Participant characteristics

The study participants were mostly male, diverse in terms of age, and most had a nursing background. They had a wide range of managerial experience, and most had experience with PHEC fieldwork prior to assuming their managerial positions (Table 1).

**Table 1:** Participant characteristics

**Managers’ age/min = 41/max = 65 (Years)**	
41–44	4
45–54	7
55–65	4
Average age	50
**Managers’ gender**	
Male	11
Female	4
**Time as manager/min = 1/max = 34 (Years)**	
1–2	2
3–7	5
8–16	6
> 17	2
Average time as manager	10
**Managers’ basic profession**	
Nurse (RN)	13
Paramedic	2
**PHEC field work experience**	
Yes	14
No	1

### Experiences leading prehospital emergency care

Three generic categories were identified in this study: challenges to the managerial role, openness and trust, and experience-based leadership (Fig. 1).

### Challenges to the managerial role

A lack of physical proximity increased academic competence, variations in the degree of staff responsibility and independence, and their impact on work culture created challenges to the managerial role.

#### A lack of physical proximity created psychosocial distance between staff and managers

Managers described that a lack of physical proximity in the work environment created psychosocial distance between staff and managers, mainly because the staff’s work with patients was conducted far away from the central workplace itself but also because the staff´s schedule, involved a large proportion of the staff working irregular hours around the clock. Some managers described challenges created by responsibility for several units, geographic separation, or when they needed to attend meetings in places other than their own workplace. Working in different physical places reduced the possibility of personal meetings and impeded working relationships between managers and staff. Large staff groups were also described as contributing to increased psychosocial distance between managers, individual staff, and the group. The greater the physical distance was, the more difficult it was for managers to promote psychological well-being, encourage engagement, increase participation, and support individuals in taking responsibility. Managers mentioned that they usually refrained from communicating with staff while they provided patient care, mostly because of a lack of physical proximity in the way work was structured but also because they lacked the time to participate in the care process:

*“In fact, as a manager in the ambulance service, you are constantly absent, in the sense that you do not participate in the care process*” (P02).

Managers mentioned how their presence facilitated problem-solving and decision-making. Communication, support, and participation were considered crucial to the well-being of individuals, especially when their physical presence was limited. Written communication, used as a substitute for face-to-face interaction, was likely to lead to confusion. The use of technology such as Microsoft Teams to communicate was seen as complementary and not equivalent to physical presence and face-to-face dialogue.

#### The increased academic competence of staff generated new demands on leadership

Managers experienced challenges related to ever-increasing academic competence and staff need for knowledge, the division of responsibilities within the work team and trust in informal practices. Staff, especially nurses (RNs), were often highly educated, with master's degrees and well-developed analytical skills. The RNs had both breadth and depth in their competencies. Many had specialist knowledge and were described as ambitious and in continual pursuit of stimulating tasks and opportunities for continued skills development. Differences in staff competence levels, for example, between RNs with or without specialist training, had implications for competence development, responsibility-taking, job complexity, the ability to self-organize, and the desire to continually improve. Decisions about role allocation and responsibilities were often made in real time on the basis of patients’ needs, with tasks distributed to ensure effective care. Some managers felt that it was challenging to address this desire for continued professional (academic) development. They described nurses as independent and strong-willed, which led to managerial challenges:

*“Nurses, they are a special group to deal with. In regard to information and cooperation, they are difficult to control. At the same time, they often take initiative and are very forward-thinking”* (P04).

#### Variations in the degree of staff responsibility and independence influenced compliance with procedures and guidelines, which in turn affects work culture

Managers confirmed how staff responsibility and independence affected compliance with procedures and guidelines. The need for commitment and participation by both managers and staff was emphasized. Direct control of work was undesirable. A balanced leadership approach, blending authority with care, seemed to be most appreciated. Staff were expected to participate in workplace meetings; take responsibility for following rules, procedures, and guidelines; and manage information and communication. When this was not the case, managers felt that they needed to take on the role of encouraging, supporting and ensuring that staff took responsibility:

*“When personal responsibility does not truly work, then you must help them along”* (P06).

Managers reflected on the importance of creating a culture of participation to support a positive and sustainable work environment. However, staff could sometimes negatively impact workplace culture through a lack of self-regulation. To counteract this, managers worked to create a positive and open work climate and a supportive team culture, with opportunities for involvement and encouraging staff to lead and support each other. By these means, positive norms could be established.

### Openness and trust

Open, direct, and personalized communication; supportive relationships; authenticity and empathic ability; engendered openness; and trust.

#### Open, direct and personalized communication created clarity

Managers also reflected on communication, with a preference for an open and transparent style. Managers mentioned that one-way communication (information dissemination) was more commonly used and preferred by some staff than two-way closed-looped communication (dialogue). However, dialogue was desired by managers, who cited its exploratory nature and potential to create understanding and provide support and feedback on the information given. Despite this preference, dialogue was considered difficult to conduct due to a lack of physical proximity:

*“How can I communicate more clearly? What do I, as individuals, need to think about when I communicate?”* (P15).

Although some of the staff preferred clear-cut and direct one-way communication, it was not always easy to communicate this way, even if it could reduce or avoid confusion. Managers felt that it was necessary to have the opportunity to meet the staff’s questions and adapt the communication style to suit the individual or group and the situation to ensure a clear understanding of the message and therefore consulted and collaborated with others to refine the message before it was presented.

#### Supportive relationships created psychological well-being, inspired commitment and contributed to increased participation

Managers saw staff well-being as central to a well-functioning working group. A supportive atmosphere and good relationships were considered requisite for an attractive workplace, which in turn contributed to job satisfaction. Managers sought to create a climate where managers and employees could collaborate, support, and listen to one another to share constructive feedback, including work well done. Managers saw this as the path to inspire commitment and create participation.

Managers felt that coaching, which involved asking questions and providing support and guidance, was a method that prevented problems from occurring and reinforced progress. They described how coaching created security and contributed to increased responsibility and initiative-taking, which eventually improved patient care.

“[As manager] *I am more of a coach. I am more the one who … creates the conditions for staff to do a good job for our patients”* (P02).

Managers emphasized that staff well-being at work extended beyond the boundaries of the workplace due to the interconnectedness of professional and private life. Therefore, they chose to take a holistic view of staff well-being, support, and assistance by offering advice, even of a private nature.

Outside the leadership sphere, managers listed psychosocial support functions for individuals within team constellations, such as peer support and mentoring. Managers considered these functions to be important complements to their own leadership for the creation of individual well-being and a secure work environment.

#### Authenticity and empathy strengthened interpersonal relationships

Authenticity, empathy, and transparency were considered important for building trust between managers and staff. Managers felt that it was important to support a positive work environment by being available, listening to staff, and acting quickly to solve problems. They saw a collaborative and supportive atmosphere as crucial to creating an attractive workplace.

Managers defined authenticity as being true to whom they presented themselves as being and aware of their own intentions. Openness about mistakes also built trust. Maintaining good relationships with individuals and validating individuals’ efforts were considered essential to sustaining job satisfaction. Social exchanges and relaxed dialogue created a collaborative atmosphere. Transparency, responsiveness, and empathy, as well as explaining the “why” behind actions, were considered crucial to building trust, engagement, and confidence among staff and managers:

*“The way we managers are, is so incredibly important because it reflects on everything”* (P10).

### Experience-based leadership

Practical applications and reflection; theoretical training combined with real-world practice; support from managers and peers; and supportive organisational structures developed experience in the managerial role.

#### Leadership was cultivated over time through practical application and reflection

Managers found the managerial role challenging. They mentioned that when they persevered, structures would eventually appear, problems would become solvable, and both good and bad experiences would contribute to personal development. Skills acquisition over time engendered a sense of security in the role. This increased managers’ self-trust in their own capabilities and resulted in a clearer understanding of their duties.

Managers described that developed practical application over time prepared them to address new situations they had not previously encountered. Managers learned from their mistakes, conflicts they dealt with, and regrets about what they wished they had done better:

*“At first, you’re quite wobbly, when you haven’t got a lot of experience. Of course, over time you learn, making mistakes that you then do not repeat”* (P02).

Managers felt that taking time for reflection on and discussion about their own work performance was crucial to improve supervisory processes and cooperation and to promote individual and collective development. They considered how their knowledge of tasks and workflows in the field simplified their decision-making. Their field experience helped them prioritize and instilled in them the importance of collaboration.

#### Preference for combining theoretical leadership training with real-world practice

Managers had a diverse range of experiences in terms of types, frequencies, and timings of leadership training. Regional training courses focused on structure and behaviors were most common, whereas university-based courses and programs were less common. Managers differed in their opinions about how these different leadership training activities influenced their leadership practices. The most highly regarded approach was theoretical training in management and leadership in combination with real-world practice as a manager. However, many times, managers were left to engage in their own leadership development:

*“In general, you pick out the parts of the education and training that you think you can use later in practice”* (P07).

Managers mentioned the benefits of real-world management experience prior to attending leadership training. They explained that this order and combination helped them better understand how to use the newly acquired theoretical knowledge in practice. To this end, a combination of different leadership training courses, based on evidence-based knowledge, was preferred and valued for managerial development, competencies that they in turn considered important to share with their staff.

#### Support from managers and peers increased confidence and spurred action

Managers emphasized that a positive work environment facilitated decision-making. Support from senior and peer managers provided a sense of security, which they saw as important for them to be effective in their leadership. Discussing and sharing their thoughts with colleagues and staff helped deepen their reflections and guide their actions, which increased their confidence and certainty in their decisions. An open and supportive team culture was considered necessary to spur action and improve performance:

*“It creates a sense of security to know that you have colleagues you can discuss with, or management that you can bounce ideas off”* (P14).

Managers found it beneficial to have access to structured coaching and mentorship from experienced colleagues to help them tackle challenges and make informed decisions, but not everyone utilized this opportunity. We found mentoring between managers, or between staff, but not between managers and staff.

Regular meetings, both within management teams and staff, were an effective way to understand and solve problems. Their own work‒life balance and support from home contributed to feeling more secure in their role. This and the trust staff placed in them as managers helped them feel competent. This instilled confidence in themselves and made it easier to support psychological well-being and support actions in others.

#### Supportive organizational structures contributed to orderliness

Managers thought it important that organizational structures supported what managers were expected to do, e.g., that their responsibilities were reinforced with decision-making mandates. They also appreciated the clarity of their frames and boundaries of their role. The establishment of these conditions and frameworks was considered a prerequisite for managerial action, which engendered a sense of clear expectations. Managers considered it important to be friendly, supportive, and inclusive while maintaining their authority as leaders. Delegating decision-making was a way to include staff and invite them to a collective decision-making process.

The managers thought it was good to strive for common goals and work for a clear organisational structure, as they believed these goals built a foundation of unity, security, and order and clear expectations for staff:

*“It generates a sense of security for them, and for me as well, that I know that … this is what we need to address”* (P10).

Aligning formal management tasks with actual operational needs was considered crucial for a well-functioning operation. However, managers’ approaches to strategic governance appeared to vary widely. Some prioritized tasks and others set and pursued objectives, whereas others do not use any strategy at all. Nevertheless, controlling, providing feedback, clarifying decisions, and creating an environment for reflection and feedback were strategies often mentioned. Managers deemed it important to function as facilitators to ease care assignment for staff to achieve operational excellence.

### Main category: the managerial role of PHEC was location independent, characterized by openness and trust, and cultivated through experience

The managerial role was location-independent, i.e., it was not connected to a physical location or physical presence in a workplace. This created leadership challenges in terms of leading strong-willed, independent, and competent individuals; addressing staff need of knowledge and development; meeting organizational requirements; and supporting a sustainable and positive work culture.

The managerial role was characterized by openness and trust, seemingly achieved through open and personalized communication, trusting relationships, authenticity, and empathy. The presence of trusting relationships seemed, in turn, to be a prerequisite to the cultivation of psychological well-being, encouraged commitment, and enhanced participation.

The managerial role was developed through experience, where theory was combined with real-world practical application, discussions, and reflection. The role appeared to be facilitated by sharing experiences and receiving feedback between managers and staff within the frames and boundaries of organisational structures.

## Discussion

This study aimed to explore managers' experiences of management and leadership in PHEC in Sweden. We found the managerial role to be location independent, with considerable work distances existing within this particular area of care. The role of manager was characterized by openness and trust, developed through open and personalized communication, trusting relationships, authenticity, and empathy. It was experienced-based and cultivated over time through practical application, discussions, and reflection.

### A location-independent managerial role

We found that managers played a *location-independent managerial role*. This role was influenced by the lack of physical proximity to the work environment where the job to be done was performed. This required managers to manage individuals and teams in an effective, trustworthy way. This physical distance led to psychological, structural, and functional distances, which corroborates previous research [37]. The physical distance described in this study was linked to the staff's remote work, to their irregular working hours, responsibilities for geographically separated units and to the managers' own presence (or absence). Research suggests that psychological distance is about beliefs, values, power, perceived similarity, and attitudes [37]. In this study, psychological distancing was linked to the social aspects of leading strong-willed, independent, and committed staff, their willingness, and the understanding of their need for knowledge and academic competence development. Structural distance is about physical, organizational, and supervisory empowerment, and functional distance is about relationships between leaders and followers, follower influence, and being "in-group" or "out-group" [37]. Here, we identified structural distances related to organizational structures, large staff groups, job distributions and role descriptions, how leadership was enacted, and managers' and staff responsibility to follow routines and guidelines. We identified functional distances in the form of culture and climate (well-being), commitment and participation, collaboration, and the individual's ability to take initiative and self-regulate, as well as feeling empathy for staff well-being and coaching them to lead and support each other through positive norms.

Communication and collaboration, as well as the use of less control and more confidence-building activities, are important elements in managing distance in a workplace [38]. This is consistent with how managers managed the work distances, where managers work with personalized communication and supportive relationships, openness (transparency and accessibility) and trust-building activities (authenticity and emphasis). Openness and trust, in particular, seemed to be key factors in managing and compensating for the distances between managers and employees in the context of PHEC. This raises further questions about when and how trust supports (or does not support) work at these distances, in which specific situations, and to what extent.

### A managerial role characterized by openness and trust

The managerial role was characterized by openness and trust, preferably promoted through a personalized and situational communication style, trusting relationships, authenticity, and empathy. Both management communication and interpersonal relationships have been shown to have a significant effect on performance and work and are best adapted to the individual and the situation [39]. The challenges that counteract communication are a lack of time, knowledge gaps and staff work schedules [39]. Other factors that limit effective communication are a reluctance to improve communication and the leader's unwillingness to take responsibility for it [40].

Relational leadership is about how leadership arises through interactions and social processes [41]. Person-centered interactions between managers and individual staff members contribute to higher-quality relationships and convey a genuine concern for individuals, which in turn can improve the quality of communication, relationships, and job satisfaction [42]. We found that managers believed in supportive and trusting relationships between themselves and staff that were based on the authenticity and interactions that demonstrated their empathic skills.

Authenticity has been shown itself to be a key factor in building trusting relationships, increasing follower engagement, and creating sustainable performance [43–46]. We identified authenticity, defined as being true to who you say you are, being aware of your intentions, and displaying empathy by caring for your staff, as primary factors that fostered trust and engagement. Empathy is considered an important aspect of leadership [47], and leaders' empathy and ethical values are linked to leadership effectiveness [48]. Frei and Morriss (2021) noted that "people tend to trust you when they think they are interacting with the real you (authenticity), when they trust your judgment and competence (logic), and when they think you care about them (empathy)" [49]. In addition, we identified factors such as availability, transparency, and responsiveness.

### A managerial role on the basis of experience

There is evidence that leadership skills are developed through experience [50, 51]. However, learning-by-doing is not an effective approach if it is not coupled with reflection and is preferably guided by mentors or peer reflection [52]. The goal of experience-based leadership development is to help managers theorize on the basis of their experiences to develop insights into what it takes to lead, to grow as a leader, and to cultivate enriching experience in colleagues, superiors, and subordinates [51]. With the myriad of offerings available to develop leadership skills, experience supported by appropriate guidance and support seems to be the most appreciated [53], as we found in our study.

Coaching can improve the process of moving through the stages of experiencing, reflecting, thinking, and acting [54]. Coactive coaching that focuses on the self can build self-confidence and self-leadership, which can enhance team and service performance [55]. We found coaching and mentoring beneficial for managers' self-development and as support and guidance for staff to engender role-confidence, deal with problems, and achieve goals. While structured coaching and mentoring training was available, where coaching was in practice neither structured, formalized, nor adapted to the PHEC-context. Mentoring was found partly structured, mostly between colleagues, but also sometimes included in supervision situations.

The importance of degree programs is limited unless they are used in conjunction with practical applications [50]. Skills in the field are taught through informal mentoring and on-the-job training [56]. The findings of the study support training coupled with practical application, as well as a focus on shared and collaborative leadership, communication and collaboration skills, emotional intelligence, and interprofessional communication [57]. However, we still need to better understand what and when a specific manager will learn from a specific experience [58]. The challenge is to find the right person to provide the right experience to learn from [59]. Otherwise, leaders may learn the wrong things or just not do the right things.

### Implications for practice

The findings suggest mangers should focus on planning and structuring how they embody their leadership, to meet the challenges of physical distance, job distributions, role descriptions, competence development, resources and staffing, and work interventions.

Of the leadership practices often described within the context of health care, authentic [12], adaptive [13], collaborative [14], servant [15–17], and supportive leadership [18] styles appeared most aligned to the work in PHEC. Our findings suggest a leadership focus on encouraging well-being through commitment, participation, and collaboration in teams and coalitions. Key competencies that help staff take a larger role in the care mission include authenticity, adaptiveness, facilitation, openness, accessibility, transparency, and empathy.

To improve consistency in managerial development, training should be adapted to context, coupled with practical application, guided by principles, include mentors or peer reflection, focus on authentic, adaptive, shared, collaborative, servant, and supportive leadership.

### Methodological limitations

The study’s transferability is limited due to the contextual anchoring of the findings. However, we suggest that there is an adequacy (credibility) in the findings and that, through the choice of study design, we have interpreted (confirmability) the data in a stable and repeatable (dependability) manner, as far as possible, through iterative analysis and revision of the findings with the involvement of all the authors. We also strove to develop thick descriptions of the phenomena to improve the transferability of the findings to other contexts [60, 61].

One of the researchers had managerial oversight over some of the interviewed managers. To mitigate this dependency and potential biases, special attention was given to the information and consent process [62]. For these managers, a researcher unaffiliated with them conducted the interviews. Reflexivity exercises were also conducted to find and expose, to circumvent, potential biases in the analysis and reporting of the data. We have thus striven to address this dependency in such a way that it should have no significant impact on the findings.

## Conclusion

In this study, we explored managers’ experiences, and their understanding of their work, roles, and operational routines in the context of prehospital emergency care (PHEC) in Sweden.

We found the managerial role to be location independent, characterized by openness and trust, and cultivated through experience. In an environment characterized by academic training and work at distances, leaders manage individuals and remote teams while respecting individuals’ independence. They supported staff competence development and their desire to take responsibility through open and trusting relationships established through creating opportunities for competency development and a “learning-by-doing” epistemology built upon reflective practice.

However, what managers perceive could differ from what is needed, which is why it is vital to broaden our insight, e.g., through staff interviews to gain a more complete understanding of how managerial work can be developed to improve patient centered PHEC. We therefore suggest further exploration of this topic to improve our understanding of this particular managerial role by complementing staff perceptions of management and leadership in PHEC.

## Supplementary Information

Supplementary Material 1.

Supplementary Material 2.

## Data Availability

The datasets used and/or analysed during the current study are available from the corresponding author upon reasonable request.

## Abbreviations

PHEC: Prehospital Emergency Care
P01-15: Participant 01–15
RN: Registered nurse

## References

[1] Coles E, Anderson J, Maxwell M, Harris FM, Gray NM, Milner G, et al. The influence of contextual factors on healthcare quality improvement initiatives: a realist review. Syst Rev. 2020;9(1):94. 32336290 10.1186/s13643-020-01344-3 PMC7184709

[2] Powell BJ, Mandell DS, Hadley TR, Rubin RM, Evans AC, Hurford MO, et al. Are general and strategic measures of organizational context and leadership associated with knowledge and attitudes toward evidence-based practices in public behavioral health settings? A cross-sectional observational study. Implement Sci. 2017;12(1):64. 28499401 10.1186/s13012-017-0593-9 PMC5429548

[3] Li SA, Jeffs L, Barwick M, Stevens B. Organizational contextual features that influence the implementation of evidence-based practices across healthcare settings: a systematic integrative review. Syst Rev. 2018;7(1):72. 29729669 10.1186/s13643-018-0734-5 PMC5936626

[4] Battilana J, Gilmartin M, Sengul M, Pache A-C, Alexander JA. Leadership competencies for implementing planned organizational change. Leadersh Q. 2010;21(3):422–38.

[5] Phung VH, Essam N, Asghar Z, Spaight A, Siriwardena AN. Exploration of contextual factors in a successful quality improvement collaborative in English ambulance services: cross-sectional survey. J Eval Clin Pract. 2016;22(1):77–85. 26303398 10.1111/jep.12438 PMC5215672

[6] Porter LW, McLaughlin GB. Leadership and the organizational context: Like the weather? Leadersh Q. 2006;17(6):559–76.

[7] Oc B. Contextual leadership: A systematic review of how contextual factors shape leadership and its outcomes. Leadersh Q. 2018;29(1):218–35.

[8] Wajdi BN. The differences between management and leadership. Sinergi: Jurnal Ilmiah Ilmu Manajemen7. 2017;7(1):75–84.

[9] Lunenburg FC. Leadership versus Management: A Key Distinction—At Least in Theory. Int J Manage Bus Adm. 2011;14(1):1–4.

[10] Gilmartin MJ, D’Aunno TA. 8 Leadership Research in Healthcare. Acad Manag Ann. 2007;1(1):387–438.

[11] Bass BM. Two Decades of Research and Development in Transformational Leadership. Eur J Work Organ Psy. 2010;8(1):9–32.

[12] Wong CA, Giallonardo LM. Authentic leadership and nurse-assessed adverse patient outcomes. J Nurs Manag. 2013;21(5):740–52. 23865927 10.1111/jonm.12075

[13] Storkholm MH, Mazzocato P, Savage C. Make it complicated: a qualitative study utilizing a complexity framework to explain improvement in health care. BMC Health Serv Res. 2019;19(1):842. 31727069 10.1186/s12913-019-4705-x PMC6857274

[14] Chatterjee R, Suy R, Yen Y, Chhay L. Literature review on leadership in healthcare management. J Soc Sci Studies. 2017;5(1):38–47.

[15] Ling Q, Liu F, Wu X. Servant Versus Authentic Leadership. Cornell Hospitality Quarterly. 2016;58(1):53–68.

[16] Eva N, Robin M, Sendjaya S, van Dierendonck D, Liden RC. Servant Leadership: A systematic review and call for future research. Leadersh Q. 2019;30(1):111–32.

[17] Asamani JA, Naab F, Ofei AMA. Leadership styles in nursing management: implications for staff outcomes. Journal of Health Sciences. 2016;6(1):23–36.

[18] Dayanti PR, Eliyana A, Emur AP, Pratama AS. Supportive leadership: a literature review. Int J Sci Manag Studies (IJSMS). 2022;5(2):74–80.

[19] National Board of Health and Welfare's regulations on ambulance care, etc (SOSFS 2009:10). National Board of Health and Welfare. https://www.socialstyrelsen.se/kunskapsstod-och-regler/regler-och-riktlinjer/foreskrifter-och-allmanna-rad/konsoliderade-foreskrifter/200910-om-ambulanssjukvard-m.m/.

[20] Welfare NBoHa. Sweden's prehospital emergency care - current situation, assessment, and development proposals Stockholm; 2023. Report No.: 2023–2–8337

[21] Swedish Health and Medical Services Act (2017:30).

[22] Holmberg M, Fagerberg I, Wahlberg AC. The knowledge desired by emergency medical service managers of their ambulance clinicians - A modified Delphi study. Int Emerg Nurs. 2017;34:23–8. 28545930 10.1016/j.ienj.2017.03.007

[23] Sundstrom BW, Dahlberg K. Being prepared for the unprepared: a phenomenology field study of Swedish prehospital care. J Emerg Nurs. 2012;38(6):571–7. 22088772 10.1016/j.jen.2011.09.003

[24] Karmelic E, Lindlof H, Luckhaus JL, Castillo MM, Vicente V, Harenstam KP, et al. Decision-making on the fly: a qualitative study of physicians in out-of-hospital emergency medical services. BMC Emerg Med. 2023;23(1):65. 37286931 10.1186/s12873-023-00830-w PMC10246870

[25] The SAGE Handbook of Qualitative Research. Fifth Edition ed. Denzin NK, Lincoln YS, editors. United States of America: SAGE Publications, Inc; 2018.

[26] Kvale S, Brinkmann S. Learning the craft of qualitative research interviewing. Tredje upplagan ed: Sage Publications Inc; 2014.

[27] Elo S, Kyngas H. The qualitative content analysis process. J Adv Nurs. 2008;62(1):107–15. 18352969 10.1111/j.1365-2648.2007.04569.x

[28] Tong ASP, Craig J. Consolidated criteria for reporting qualitative research (COREQ): a 32-item checklist for interviews and focus groups. Int J Qual Health Care. 2007;19:349–57. 17872937 10.1093/intqhc/mzm042

[29] SCB Statistics Sweden. 2023.

[30] Campbell S, Greenwood M, Prior S, Shearer T, Walkem K, Young S, et al. Purposive sampling: complex or simple? Research case examples J Res Nurs. 2020;25(8):652–61. 34394687 10.1177/1744987120927206 PMC7932468

[31] Palinkas LA, Horwitz SM, Green CA, Wisdom JP, Duan N, Hoagwood K. Purposeful Sampling for Qualitative Data Collection and Analysis in Mixed Method Implementation Research. Adm Policy Ment Health. 2015;42(5):533–44. 24193818 10.1007/s10488-013-0528-y PMC4012002

[32] Strauss A, Corbin J. Basics of Qualitative Research: Techniques and procedures for developing grounded theory. SAGE Publications. 1998.

[33] Low J. A Pragmatic Definition of the Concept of Theoretical Saturation. Sociol Focus. 2019;52(2):131–9.

[34] Cash P, Snider C. Investigating design: A comparison of manifest and latent approaches. Des Stud. 2014;35(5):441–72.

[35] Yaffe P. How to Properly Use Quotations. Ubiquity - Association for Computing Machinery. 2023;2:1–8.

[36] Eldh AC, Årestedt L, Berterö C. Quotations in qualitative studies: reflections on constituents, custom, and purpose. Int J Qualitative Methods. 2020;19:1609406920969268.

[37] Antonakis J, Atwater L. Leader distance: a review and a proposed theory. Leadersh Q. 2002;13:673–704.

[38] Hurmekoski M, Haggman-Laitila A, Lammintakanen J, Terkamo-Moisio A. Nurse leaders’ experiences of remote leadership in health care. Leadersh Health Serv (Bradf Engl). 2023;36(4):579–94. 10.1108/LHS-01-2023-0003 PMC10853847 37144970

[39] Nordby H. Experienced Challenges in Prehospital Management: Communication and Cooperation in Manager-Employee Interaction. J Health Manag. 2015;17(1):98–117.

[40] Hicks JM. Leader Communication Styles and Organizational Health. Health Care Manag (Frederick). 2020;39(4):175–80. 33079769 10.1097/HCM.0000000000000305

[41] Uhl-Bien M. Relational Leadership Theory: Exploring the social processes of leadership and organizing. Leadersh Q. 2006;17(6):654–76.

[42] Fix B, Sias PM. Person-Centered Communication, Leader-Member Exchange, and Employee Job Satisfaction. Commun Res Rep. 2006;23(1):35–44.

[43] Alkaabi OA. Relationships Among Authentic Leadership, Manager Incivility and Trust in the Manager. 2018.

[44] Alilyyani B. The Effect of Authentic Leadership on Nurses’ Trust in Managers and Job Performance: A Cross-Sectional Study. Nurs Rep. 2022;12(4):993–1003. 36548168 10.3390/nursrep12040095 PMC9784480

[45] Walumbwa FO, Avolio BJ, Gardner WL, Wernsing TS, Peterson SJ. Authentic Leadership: Development and Validation of a Theory-Based Measure†. J Manag. 2007;34(1):89–126.

[46] Avolio BJ, Walumbwa FO, Weber TJ. Leadership: current theories, research, and future directions. Annu Rev Psychol. 2009;60:421–49. 18651820 10.1146/annurev.psych.60.110707.163621

[47] Holt S, Marques J. Empathy in Leadership: Appropriate or Misplaced? An Empirical Study on a Topic that is Asking for Attention. J Bus Ethics. 2011;105(1):95–105.

[48] Mahsud R, Yukl G, Prussia G. Leader empathy, ethical leadership, and relations-oriented behaviors as antecedents of leader-member exchange quality. J Manag Psychol. 2010;25(6):561–77.

[49] Frei F, Morriss A. Trust: The Foundation of Leadership. Lead Lead. 2020;2021(99):20–5.

[50] McCall MW. Recasting Leadership Development. Ind Organ Psychol. 2015;3(1):3–19.

[51] Thomas RJ, Cheese P. Leadership: experience is the best teacher. Strategy & Leadership. 2005;33(3):24–9.

[52] Savage M, Storkholm MH, Mazzocato P, Savage C. Effective physician leaders: an appreciative inquiry into their qualities, capabilities and learning approaches. BMJ Leader. 2018;2(3):95–102.

[53] West M, Armit K, Loewenthal L, Eckert R, West T, Lee A. Leadership and Leadership development in Health Care: The Evidence Base. Faculty of Medical Leadership and Management The King´s Found 2015.

[54] Kolb AY, Kolb DA. The Learning Way Meta-cognitive Aspects of Experiential Learning. SAGE Publications. 2009;40(3):297–327.

[55] Cable S, Graham E. “Leading Better Care”: An evaluation of an accelerated coaching intervention for clinical nursing leadership development. J Nurs Manag. 2018;26(5):605–12. 29600826 10.1111/jonm.12590

[56] Leggio WJ Jr. The State of Leadership Education in Emergency Medical Services: A Multi-national Qualitative Study. Prehosp Disaster Med. 2014;29(5):478–83. 25156075 10.1017/S1049023X14000867

[57] Jankelová N, Joniaková Z, Romanová A. The need for management education of healthcare management employees. Int J Health Plann Manage. 2002;37(1):301–17. 10.1002/hpm.3325 34585433

[58] Hezlett SA. Suggestions for New Research on Experience-Based Learning. Ind Organ Psychol. 2015;3(1):56–60.

[59] McCall M. Leadership development through experience. Academy of Management Executive. 2004;18(3):127–30.

[60] Tracy SJ. Qualitative Quality: Eight “Big-Tent” Criteria for Excellent Qualitative Research. Qual Inq. 2010;16(10):837–51.

[61] Guba EG. Criteria for Assessing the Trustworthiness of Naturalistic Inquiries. ERIC/ECTJ Annual Review Paper. 1981;29(2):75–91.

[62] World Medical Association. WMA Declaration of Helsinki - Ethical principles for medical research involving human subjects. 2024.

[63] Law (Sfs 2003:460) Concerning The Ethical Review Of Research Involving Humans. 2003.

[64] Regulation (EU) 2016/679 of the European Parliament and of the Council of 27 April 2016 on the protection of natural persons with regard to the processing of personal data and on the free movement of such data, and repealing Directive 95/46/EC (General Data Protection Regulation). 2016.

