# Supplementary material for: Location-independent leadership: managers’ experiences leading prehospital emergency care in Sweden – a qualitative study
Source: BMC Health Serv Res. 2025 Feb 17;25:265. doi: 10.1186/s12913-025-12433-1 (PMC11834692; doi:10.1186/s12913-025-12433-1)
Supplement: Supplementary file 2 — Supplementary Material 2. [file 12913_2025_12433_MOESM2_ESM.pdf]

Interview guide – Location-independent leadership: managers' experiences leading prehospital emergency care in Sweden – a qualitative study

The interview was developed for this study and has not been published elsewhere.

The data were collected through in-depth one-to-one interviews with open-ended questions.

The main interview question was “What is your experience of management and leadership in prehospital emergency care?” Follow-up questions such as "Can you tell me more?" were posed. Follow-up questions were not predetermined, they were generated during the interview.

Below are examples of follow-up questions that were generated during the first of 15 interviews.

1. How did you think it is complex? (follow-up question))

2. And I immediately become curious about this thing about remote leadership. Can you tell us more about it, how do you work with it?

3. Why is it important to you?

4. Yes. And what do you think employees feel about it?

5. Mmm. Tell me more about it...

6. Mmm. You say that there are big changes in recent years? What does this change entail, apart from increased responsibility?

7. How do you see it?

8. Yes, can you tell us more about this relationship between managers?

9. How have you managed to do that?

10. Why is it important?

11. Yes. What do you think is the challenge then? In your role?

12. How do you handle it?

13. What does this strategic leadership mean to you?

14. Yes. The knowledge, this that it moves forward, can you tell us more about it? In your role as a manager?

15. Is it important to have courage in your leadership?

16. And what is the biggest change?

17. Where does the challenge lie?

18. Mmm. Is it important to have support from your manager in your leadership?
